# Gaussian Processes and Polynomial Chaos Expansion for Regression Problem: Linkage via the RKHS and Comparison via the KL Divergence

**DOI:** 10.3390/e20030191

**Published:** 2018-03-12

**Authors:** Liang Yan, Xiaojun Duan, Bowen Liu, Jin Xu

**Affiliations:** College of Liberal Arts and Sciences, National University of Defense Technology, Changsha 410073, China

**Keywords:** Gaussian process, polynomial chaos expansion, reproducing kernel Hilbert space, Kullback–Leibler divergence, experimental design

## Abstract

In this paper, we examine two widely-used approaches, the polynomial chaos expansion (PCE) and Gaussian process (GP) regression, for the development of surrogate models. The theoretical differences between the PCE and GP approximations are discussed. A state-of-the-art PCE approach is constructed based on high precision quadrature points; however, the need for truncation may result in potential precision loss; the GP approach performs well on small datasets and allows a fine and precise trade-off between fitting the data and smoothing, but its overall performance depends largely on the training dataset. The reproducing kernel Hilbert space (RKHS) and Mercer’s theorem are introduced to form a linkage between the two methods. The theorem has proven that the two surrogates can be embedded in two isomorphic RKHS, by which we propose a novel method named Gaussian process on polynomial chaos basis (GPCB) that incorporates the PCE and GP. A theoretical comparison is made between the PCE and GPCB with the help of the Kullback–Leibler divergence. We present that the GPCB is as stable and accurate as the PCE method. Furthermore, the GPCB is a one-step Bayesian method that chooses the best subset of RKHS in which the true function should lie, while the PCE method requires an adaptive procedure. Simulations of 1D and 2D benchmark functions show that GPCB outperforms both the PCE and classical GP methods. In order to solve high dimensional problems, a random sample scheme with a constructive design (i.e., tensor product of quadrature points) is proposed to generate a valid training dataset for the GPCB method. This approach utilizes the nature of the high numerical accuracy underlying the quadrature points while ensuring the computational feasibility. Finally, the experimental results show that our sample strategy has a higher accuracy than classical experimental designs; meanwhile, it is suitable for solving high dimensional problems.

## 1. Introduction

Computer simulations are widely used in learning tasks, where a single simulation is an instance of the system [1,2]. A simple approach to the learning task is to randomly sample input variables and run the simulations for each input to obtain the features of the systems. Similar approaches are utilized in Monte Carlo techniques [3]. However, even a single simulation can be computationally costly due to its high complexity, and so, obtaining a trustworthy result via sufficient simulations becomes intractable. Mathematical methods and statistical theorems are introduced to generate surrogate models to replace the simulations, especially when dealing with complex systems with many parameters [4,5]. Although the main drawback of surrogate models is that only approximations can be obtained, they are computationally efficient whilst maintaining the essential information of the systems, hence analyzing the properties of the system. Attempting to construct surrogate models with an acceptable number of simulations necessitates the development of robust techniques to determine their reliability and validity [6,7,8]. Plenty of researchers are working on improving sampling strategies to decrease the number of simulations, which makes the task more significant [9]. With an increasing number of surrogate models being developed, there needs to be a comprehensive understanding of the uncertainties introduced by those models. The main purpose of uncertainty quantification (UQ) is to establish a relationship between input and output, i.e., the propagation of input uncertainties, and then to quantify the difference between surrogate models and original simulations. UQ can provide a measure of the surrogate model’s accuracy and an indication of how to update the model at the same time [10,11,12].

Denote *f* as a function (or simulator) of the original system, then given experimental design X, the output Y=f(X) is produced, where caption notation is used because the input and output are usually vectors (or matrices) in simulations. From a statistical perspective, the input uncertainties are introduced by their randomness, so we represent the input with a random variable x, whose prior probability density function (PDF) is p(x), such as the multivariate Gaussian distribution; as for the output uncertainties, a common technique is to integrate the system uncertainty and the approximation error as a noise term ϵ. In fact, the output y is also a random variable y=f(x)+ϵ determined by f,x and ϵ. Now, suppose a surrogate $\bar{f}\left(x\right)$ is constructed to approximate f(x), then UQ is used to identify the distribution and statistical features (for example, Kullback–Leibler divergence) of y, which are essential to the validation and verification of surrogates. Basically, there are two preconditions that need to be satisfied: firstly, the surrogate models are well defined, i.e., any $\bar{f}$ is a measurable function with respect to (w.r.t) corresponding probability space p(x); secondly, techniques are needed that learn from the prior information to obtain the best guess of the true function.

There is a number of studies proposing different surrogates for specific applications in the literature, such as multivariate adaptive regression splines (MARS) [13], support vector regression (SVR) [14], artificial neural network (ANN) [15] for reliability and sensitivity analyses and kriging [16] for structural reliability analysis. We mainly focus on two popular methods that have been extensively studied recently. One popular method that is extensively studied in the literature is the polynomial chaos expansion (PCE), also known as a spectral approach [17]. PCE aims to represent an arbitrary random variable of interest as a spectral expansion function of other random variables with prior PDF. Xiu et al. [18,19,20] have generalized the PCE in terms of the Askey scheme of polynomials, so the surrogates can be expressed by a series of orthogonal polynomials w.r.t the distributions of the input variables. These polynomials can be extended as a basis of a polynomial space. In general, methods used to solve PCE problems are categorized as two types: intrusive and non-intrusive. The main idea behind the intrusive methods is the substitution of the input x and output f(x) with the truncated PCE and calculating the coefficients with the help of Galerkin projection [21]. However, the explicit formation of *f* is required to compose the Galerkin system, and a specific algorithm or program is needed to solve a particular problem. It is for these reasons that the intrusive models are not widely used; non-intrusive methods have been developed to avoid these limitations [21,22]. There are two main aspects of the non-intrusive methods: one is the choice of sampling strategies, for example Monte Carlo techniques; the other one is computational approaches. These two aspects are not independent of each other: for example, if x∼N(0,1), then the Gaussian quadrature method is introduced to solve the numerical integration and X is the set of corresponding quadrature points. Another one of the more common methods in constructing surrogate models is the Gaussian process (GP), which is actually a Bayesian approach. Instead of attempting to identify a specific real model of the system, the GP method provides a posterior distribution over the model in order to make robust predictions about the system. As described in the highly influential works [23,24,25,26], the GP can be treated as a distribution over functions with properties controlled by a kernel. For the two prerequisites discussed in the previous paragraph, the GP generates a surrogate model that lies in a space spanned by kernels; meanwhile, Bayesian linear regression or classification methods are introduced to utilize the prior information.

Both the PCE and GP methods build surrogates, but there are some differences between them. The PCE method builds surrogates of a random variable y as a function of another prior random variable x rather than the distribution density function itself. The PCE surrogates are based on the orthogonal polynomial basis corresponding to the p(x), so it is simple to obtain the mean and standard deviation of y. In contrast, the GP utilizes the covariance information so that it performs better in capturing the local features. Although both the PCE and GP approaches are feasible methods to compute the mean and standard deviation of y, the PCE performs more efficiently than the GP method.

As mentioned above, both the PCE and GP methods have their own trade-offs to consider when building surrogates, and there exists a connection to be explored. According to Paul Constantine’s work [27], ordinary kriging (i.e., GP in geostatistics) interpolation can be viewed as a transformed version of the least squares (LS) problem, and the PCE can be viewed as the least squares with selected basis and weights. However, the GP reverts to interpolation when the noise term is zero. When taking the noise term into consideration, the Gaussian process with the kernel (i.e., covariance matrix) $X^{T}X$ can be viewed as a ridge regression problem [28] with a regularization term. Furthermore, different numerical methods can affect the precision of the PCE method, as well. For example, Xiu [20] analyzed the aliasing error w.r.t the projection method and interpolation method. Thus, the inherent connection of the two models cannot be simply summarized as an LS solution, and how to output a model with high precision remains an interesting question.

There are connections between the PCE and GP methods that have been explored by R. Schobi, etc. They introduced a new meta-modeling method naming PC-kriging [29] (polynomial-chaos-based kriging) to solve the problems like rare event estimation [30], structural reliability analysis [31], quantile estimation [32], etc. In their papers, the PCE models can be viewed as a special form of GP where a Dirac function is introduced as the kernel. They also proposed the idea that the PCE models have better performance in capturing the global features and that the GP models approximate the local characteristics. We would like to describe the PC-kriging method as a GP model with a PCE-form trend function along with a noise term. The global features are dominated by the PCE trend, and local structures (residuals) are approximated by the ordinary GP process. The PC-kriging model thus introduces the coefficients as parameters to be optimized, and the solution can be derived by Bayesian linear regression with the basis consisting of the PCE polynomials. They also use the LARSalgorithms to calibrate the model and to select a sparse design. They construct a rigid framework to optimize the parameters, validate and calibrate the model and evaluate the model accuracy.

Unlike the PC-kriging, which takes the PCE as a trend, this paper focuses on the construction of the kernel in the GP to solve the regression problems, through which we can combine the two methods into a unified framework, unifying positive aspects from both and in so doing refining the surrogates. In other words, we wish to find the connection between the GP and the PCE by analyzing the attribution of their solutions, and we want to propose a new approach to achieve high-precision predictions. The main idea of this paper is described as follows. Firstly, the PCE surrogate is embedded in a Hilbert space whose bases are the orthonormal polynomials themselves, then a suitable inner product and a Mercer kernel [33] are defined to build a reproducing kernel Hilbert space (RKHS) [33]. Secondly, on the other hand, the kernel of the GP can be de-composited as the product of eigenfunctions, and we can define an inner product to generate a RKHS, as well. We have explicitly elaborated the two procedures respectively and proven that the two RKHS are isometrically isomorphic. Hence, a connection between these two approaches has been established via RKHS. Furthermore, we can obtain a solution of the PCE model by solving a GP model with the Mercer kernel w.r.t the PCE polynomial basis. We name this approach Gaussian processes on polynomial chaos basis (GPCB). In order to illustrate the capability of the GPCB method, we use the Kullback–Leibler divergence [34] to explicitly compare the PDFs of the posterior prediction of the GPCB and PCE method. Provided that the true function can be approximated by a finite number of PCE bases, it can be concluded that the GPCB can converge to the optimal subset of the RKHS wherein the true function lies.

The experimental design from the PCE model, i.e., the full tensor product of quadratures in each dimension, is used in the GPCB. We have overcome two concerns about the PCE and GP, respectively. Firstly, the PCE is based on a truncated polynomial basis, while the GPCB keeps all polynomials, which can be regarded as maintaining information in every feature. Secondly, the GP’s behavior depends on the experimental design; however, it often achieves the optimal result in local small datasets. The quadrature points derived from the PCE model are distributed evenly in the input space, and those points have high numerical precision w.r.t the polynomial basis; hence, they can work well with the GPCB. However, we must admit that the GPCB is still a GP approach, so when the dimension of input variables grows, the computational burden is on the table. In order to cope with the high dimensional problems, sampling strategies to lower the number of experimental designs are put on the table. The AK-MCSmethod [35] is a useful tool that adaptively selects new experimental designs; however, the experimental design tends to validate the selected surrogate model. We propose a new method that is model-free and that makes full use of the quadrature points. We randomly choose a sparse subset from the quadrature points to form a new experimental design while maintaining the accuracy. Several classical sampling strategies like MC, Halton and LHS are introduced to compare their capabilities. Our sample scheme has superior performance under the conditions in this paper. The GPCB is a novel method to build surrogate models, and it can be used for various physical problems such as reliability analysis and risk assessment.

This paper is divided into two parts. In Part 1, we discuss the mathematical rigor of the method: a brief summary of PCE and GP is presented in Section 2; the reproducing kernel Hilbert space (RKHS) is introduced to connect these two methods in Section 3; the GPCB method is proposed based on the discussion in Section 4; meanwhile, a theoretical Kullback–Leibler divergence between the GPCB and PCE method is demonstrated. In Part 2, an explicit Mehler kernel is presented with the Hermite polynomial basis in the last part of Section 4; several tests of the GPCB with some benchmark functions are presented in Section 5, along with the random constructive sampling method for high dimensional problems.

## 2. Brief Review of PCE and GP

Firstly, we want to have a clear idea of how the PCE and GP work under the circumstances that, e.g., only samples of input *X* and output *Y* are obtained. Different assumptions are made to cope with PCE and GP, respectively, and the processing procedures are presented in the following subsections.

### 2.1. Polynomial Chaos Expansion

Just as discussed in Section 2.2, the output is assumed to be represented by a model y=f(x)+ϵ, where f(x):x∈Ω→R is the real function underneath and $\epsilon ∼N(0,\sigma_{\epsilon }^{2})$. Here, we define $x={\left(x_{1},x_{2},\ldots ,x_{d}\right)}^{T}$ as a *d*-dimensional vector of independent random variables in a bounded domain $\Omega \subset R^{d}$. Suppose $\left\{x_{i},\;i=1,\ldots ,d\right\}$ are independent and identically distributed; the joint PDF has the form $p\left(x\right)=\prod_{i=1}^{d}p\left(x_{i}\right)$. In the context of PCE, we aim to seek a surrogate of the model f(x) as an expansion of a series of orthonormal polynomials $\phi_{\alpha }\left(x\right)$:

(1) $$\begin{array}{c} f\left(x\right)=\sum_{\alpha \in N^{d}}\beta_{\alpha }\phi_{\alpha }\left(x\right),\alpha =\left\{\alpha_{1},\ldots ,\alpha_{d}\right\} \end{array}$$

where α is the multi-index, $\phi_{\alpha }\left(x\right)=\prod_{i=1}^{d}\phi_{\alpha_{i}}^{\left(i\right)}\left(x_{i}\right)$, $\int_{\Omega_{i}}\phi_{m}^{\left(i\right)}\left(x_{i}\right)\phi_{n}^{\left(i\right)}\left(x_{i}\right)p_{i}\left(x_{i}\right)dx_{i}=\delta_{mn}$ with $\Omega_{i}$ the marginal domain of Ω and $\delta_{mn}$ the Kronecker delta. Xiu et al. [20] have summarized various correspondences between the distribution and polynomial basis to form generalized polynomial chaos.

It is proven that the original model f(x) can be approximated to any degree of accuracy in a strong sense [20], e.g., mean-square norm $∥f\left(x\right)-\sum_{\alpha \in N^{D}}\beta_{\alpha }\phi_{\alpha }\left(x\right)∥$ in an $L^{2}$ norm defined on Ω, although *f* is not necessarily the span of orthonormal polynomial bases. Since we are unable to calculate an infinite series, the truncation scheme corresponding to multi-index α is introduced such that we can rearrange the polynomials. For simplicity, we can rewrite Equation (1) in the following form:

(2) $$\begin{array}{c} f\left(x\right)≊\sum_{l=0}^{M}\beta_{l}\phi_{l}\left(x\right)≜f_{P}, \end{array}$$

We can simply solve the above system via the ordinary least squares method or the non-intrusive method. Specifically, we focus on the non-intrusive projection method, whereby we can directly obtain the coefficients by taking the expectation value of Equation (2) multiplied by $\phi_{l}\left(x\right)$:

(3) $$\begin{array}{c} \beta_{l}=\int f\left(x\right)\phi_{l}\left(x\right)p\left(x\right)dx\approx \sum_{i=1}^{N}\omega_{i}f\left(X_{i}\right)\phi_{l}\left(X_{i}\right),l=0,\ldots ,M \end{array}$$

where the second equation is derived by the numerical integration techniques, such as the Gaussian quadrature rule, and $\left\{X_{i},i=1,\ldots ,N\right\}$ and $\left\{\omega_{i},i=1,\ldots ,N\right\}$ are the corresponding nodes and weights. The integration is exact when f(x) is of polynomial complexity. Together with Equations (2) and (3)$,f_{P}\left(x\right)$ has the form:

(4) $$\begin{array}{c} f_{P}\left(x\right)\approx \sum_{l=0}^{M}\left[\sum_{i=1}^{N}\omega_{i}f\left(X_{i}\right)\phi_{l}\left(X_{i}\right)\right]\phi_{l}\left(x\right)≜\sum_{l=0}^{M}\beta_{l}\phi_{l}\left(x\right). \end{array}$$

$\left\{f\left(X_{i}\right),i=1,\ldots ,N\right\}$ remain unknown to us, and usually, they are substituted by $\left\{Y_{i},i=1,\ldots ,N\right\}$. Note $Y_{i}=f\left(X_{i}\right)+\epsilon_{i}$, so such a substitution will introduce noise into the surrogate; hence, the approximation error is neglected as a source of uncertainty.

### 2.2. Gaussian Process Regression

The analysis of the Gaussian process regression model [26] is reviewed in this section. A Gaussian prior is placed over function f(x), i.e., f(x)∼GP(m(x),k(x,x)), where *m* is the mean function and *k* is the kernel function, which is positive semi-definite bounded. More specifically, let $X\;=\;\left\{X_{i},\;i=1,\ldots ,N\right\}\in \;\Omega^{N}$ be the input data, and let $Y=\left\{Y_{i},i=1,\ldots ,N\right\}\in R^{N}$ be the output data, then we have Y=f(X)+ϵ with f(X)∼N(m(X),k(X,X)) and $\epsilon ∼N(0,\sigma_{\epsilon }^{2}I)$. Bear in mind that the mathematical expression of f(x) is implicit, so f(x) is approximated to achieve the best guess prediction $f_{G}$ in the statistical sense. With the help of Bayes’ theorem, prediction and corresponding variance at a new point x can be obtained by the following equations [36]:

(5) $$\begin{array}{c} \begin{array}{cc} p_{G}\left(f\left(x\right)|Y,X,x,\theta \right) & =N(f_{G}\left(x\right),cov\left(f_{G}\left(x\right)\right)),\\ f_{G}\left(x\right)≜E\left[f(x)|Y,X,x,\theta \right] & =K_{x}^{T}{\left[K+\sigma_{\epsilon }^{2}I\right]}^{-1}Y,\\ cov(f_{G}\left(x\right)) & =K_{\mathrm{xx}}-K_{x}^{T}{\left[K+\sigma_{\epsilon }^{2}I\right]}^{-1}K_{x}. \end{array} \end{array}$$

where $K=k\left(X,X\right)\in R^{N\times N}$ as the covariance matrix with $K_{ij}=k\left(X_{i},X_{j}\right)$ and $K_{x}=k\left(X,x\right)\in R^{N\times 1}$, $K_{\mathrm{xx}}=k\left(x,x\right)\in R$ are defined similarly. Note that Equation (5) shows that the mean value of the posterior distribution can be expressed as a linear combination of *N* kernel functions as follows:

(6) $$\begin{array}{c} f_{G}\left(x\right)=\sum_{i=1}^{N}\alpha_{i}k\left(X_{i},x\right),\;\alpha ={\left(K+\sigma_{\epsilon }^{2}I\right)}^{-1}Y \end{array}$$

## 3. Links between the PCE and GP

The basic concepts of PCE and GP are discussed in Section 2. GP generates a surrogate based on Bayes’ theorem and the Gaussian hypothesis; however, it is controlled by the kernel function and the experimental design and usually does not utilize prior distribution information; PCE substitutes the model with orthonormal polynomials, which is more computational efficient, but performs badly when facing noisy or big data. This section aims to build a connection between PCE and GP; hence, they can be studied in the same structure and be combined to improve the performance of the surrogates. The reproducing kernel Hilbert space will be of great help to build such a bridge, and we are going to present it as follows.

### 3.1. Generate an RKHS from a Mercer Kernel Constructed by the PCE Basis

We have obtained a complete orthonormal basis $\left\{\phi_{l}\right\}$ of Hilbert space $H:=span\{\phi_{l}\left(x\right)\}$ with inner product <f(x),g(x)>=∫f(x)g(x)p(x)dx in Section 2.1. We can see that the PCE method generates surrogates, which are actually a linear combination of $\left\{\phi_{l}\right\}$, so there exists a unique expansion $f=\sum_{l}f_{l}\phi_{l}\in H$. According to Mercer’s theorem [33], we aim to define a kernel having the following form:

(7) $$\begin{array}{c} k\left(x,x^{\prime }\right)=\sum_{l}\lambda_{l}\phi_{l}\left(x\right)\phi_{l}\left(x^{\prime }\right)\;s.t.\;k\left(x,x\right)<\infty \;for\;\forall x\in \Omega . \end{array}$$

If we have positive weights $\lambda_{l}$ that satisfy $\sum_{l}\lambda_{l}\phi_{l}^{2}\left(x\right)<\infty$, then for any x∈Ω, together with the Cauchy–Schwarz inequality, we have:

(8) $$\begin{array}{c} \left|f\left(x\right)\right|\leq \sqrt{\sum_{l}\frac{<f\left(x\right),\phi_{l}\left(x\right){>}^{2}}{\lambda_{l}}}\sqrt{\sum_{l}\lambda_{l}\phi_{l}^{2}\left(x\right)}. \end{array}$$

|f(x)| is point-wise bounded because f(x)∈H for any x∈Ω. By checking the right side of the above inequality, the second term is ensured in advance, then f(x) lies in a subspace of H such that:

(9) $$\begin{array}{c} H_{P}=\left\{f\in H\;|<f,f{>}_{H_{P}}=\sum_{l}\frac{<f\left(x\right),\phi_{l}\left(x\right){>}^{2}}{\lambda_{l}}<\infty ,\sum_{l}\lambda_{l}\phi_{l}^{2}\left(x\right)<\infty \right\}. \end{array}$$

**Proposition** **1.** *$H_{P}$ defined in Equation (9) is an RKHS with Mercer kernel defined in Equation (7).*

### 3.2. Generate an RKHS from the Reproducing Kernel Map Construction

We aim to compose a space of functions in which all the GP surrogates are embedded. Given Equation (6) and an arbitrary experimental design X, define a space of functions as follows:

(10) $$\begin{array}{c} H_{G}^{\prime }=\left\{f\left(x\right)=\sum_{i=1}^{N}f_{i}k\left(x,X_{i}\right)|N\in N,X\in \Omega^{N},x\in \Omega ,f_{i}\in R,\sum_{i=1}^{N}\sum_{j=1}^{N}f_{i}f_{j}k\left(X_{i},X_{j}\right)<+\infty \right\}. \end{array}$$

**Proposition** **2.** $H_{G}^{\prime }$ is a pre-Hilbert space with the inner product $<\cdot ,\cdot {>}_{H_{G}^{\prime }}$

Now that $H_{G}^{\prime }$ is a pre-Hilbert space and given the norm ${∥f\left(x\right)∥}_{H_{G}^{\prime }}=\sqrt{<f\left(x\right),f\left(x\right){>}_{H_{G}^{\prime }}}$, we can define a closure of $H_{G}^{\prime }$ as $H_{G}$ derived by the classical Hilbert space theory. This is an abstract space where the norm of $H_{G}^{\prime }$ extends to the closure $H_{G}$. Thus, we have a Hilbert space $H_{G}$.

**Proposition** **3.** *$H_{G}$ defined above is the unique RKHS of the kernel k(·,·).*

### 3.3. Reproducing Kernel Hilbert Spaces as a Linkage

$H_{P}$ and $H_{G}$ are RKHS with the Mercer kernel and GP kernel, respectively. We are going to investigate the relationship between the two RKHS, by which we can discuss the two approaches in a unified structure. Let X be a sample set and GP kernel k(·,·) be a real positive semi-definite kernel, then according to Mercer’s theorem, $k(X_{i},X_{j})$ has an eigenfunction expansion:

(11) $$\begin{array}{c} k\left(X_{i},X_{j}\right)=\sum_{l}\lambda_{l}\phi_{l}\left(X_{i}\right)\phi_{l}\left(X_{j}\right), \end{array}$$

where the eigenfunctions $\left\{\phi_{l}\right\}$ are orthonormal, i.e., $<\phi_{l},\phi_{l^{\prime }}>=\int \phi_{l}\left(x\right)\phi_{l^{\prime }}\left(x\right)p\left(x\right)dx=\delta_{ll^{\prime }}$ and $\left\{\lambda_{l},\phi_{l}\right\}$ satisfies $\sum_{l}\lambda_{l}\phi_{l}^{2}\left(x\right)<\infty$. Let $f_{X}\left(x\right)\in H_{G}$ with experimental design X, then we can rewrite it according to Equations (10) and (11):

(12) $$\begin{array}{c} f_{X}\left(x\right)=\sum_{i=1}^{N}f_{i}\sum_{l}\lambda_{l}\phi_{l}\left(X_{i}\right)\phi_{l}\left(x\right)=\sum_{l}\left[\lambda_{l}\sum_{i=1}^{N}f_{i}\phi_{l}\left(X_{i}\right)\right]\phi_{l}\left(x\right)≜\sum_{l}c_{l}\left(X\right)\phi_{l}\left(x\right). \end{array}$$

where $c_{l}\left(X\right)$ is identically determined by *l* and X, and it has a similar form as a function lies in $H_{P}$. Actually, given $f_{X}\left(x\right),g_{X^{\prime }}\left(x\right)\in H_{G}$, we have:

(13) $$\begin{array}{cc} <f_{X}\left(x\right),g_{X^{\prime }}\left(x\right){>}_{H_{G}} & =\sum_{i=1}^{N}\sum_{j=1}^{N^{\prime }}f_{i}g_{j}\left[\sum_{l}\lambda_{l}\phi_{l}\left(X_{i}\right)\lambda_{l}\phi_{l}\left(X_{j}^{\prime }\right)/\lambda_{l}\right]\\ & =\sum_{i}\left\{\left[\lambda_{l}\sum_{i=1}^{N}f_{i}\phi_{l}\left(X_{i}\right)\right]\left[\lambda_{l}\sum_{j=1}^{N^{\prime }}g_{j}\phi_{l}\left(X_{j}^{\prime }\right)\right]\right\}/\lambda_{l}\\ & =\sum_{l}c_{l}\left(X\right)c_{l}\left(X^{\prime }\right)/\lambda_{l}\\ & =\sum_{l}<f_{X}\left(x\right),\phi_{l}><g_{X^{\prime }}\left(x\right),\phi_{l}>/\lambda_{l}\\ & =<f_{X}\left(x\right),g_{X^{\prime }}\left(x\right){>}_{H_{P}}. \end{array}$$

The above equation gives us the information that their inner product stands in $H_{P}$, as well, so we can conclude that $f_{X}$ lies in $H_{P}$. It also shows us that the two inner products are equivalent. Next, we are going to propose a rigid theorem to prove that the two spaces are isometrically isomorphic.

**Theorem** **1.** The reproducing kernel Hilbert space $H_{G}$ of a given kernel k is isometrically isomorphic to the space $H_{P}$.

**Figure 1:**
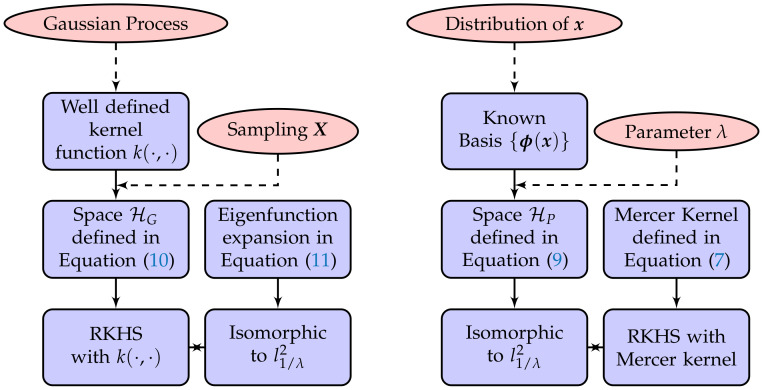
Left: Generate the reproducing kernel Hilbert space (RKHS) with the reproducing kernel map; right: generate the reproducing Mercer kernels with the polynomial chaos expansion (PCE) basis.

According to the proof of Theorem 1 in Appendix A.4, it is reasonable to introduce a weighted $l^{2}$ space $l_{1/\lambda }^{2}$ because it is difficult to find a direct linear map between $H_{G}$ (where $c_{l}$ varies according to X and *k*) and $H_{P}$ (where $c_{l}$ varies according to the distribution of x). Figure 1 shows two flowcharts used to describe different processes in generating the RKHS.

Furthermore, the GP prediction is a combination of the kernel functions, which consist of infinite eigenfunctions, while the PCE prediction is always a combination of finite polynomial bases. The Kullback–Leibler divergence (KL divergence) is a useful criterion to indicate the performance of different surrogate models. We are going to present the comparison of the GPCB and PCE methods with the help of KL divergence in the next section.

## 4. Gaussian Process on Polynomial Chaos Basis

$H_{G}$ and $H_{P}$ are isomorphic as discussed in previous Section 3, so it is natural to come up with the idea that GP can be conducted with k(·,·) as the Mercer Kernel generated by polynomial basis in the PCE, and the new model is called Gaussian process on polynomial chaos basis (GPCB). In fact, the GPCB generates a PCE-like model, but with a different philosophy. Note that the posterior distribution of the predictions regarding experimental design {X,Y} can be calculated analytically, so we are able to compute the KL divergence as well, which are presented as follows.

### 4.1. Comparison of the PCE and GPCB with the Kullback–Leibler Divergence

The true distribution of the system is always implicit in practice. Without loss of generality, the underlying true system is assumed to be $f_{\bar{P}}\left(x\right)=\sum_{l=0}^{\bar{M}}{\bar{\beta }}_{l}\phi_{l}\left(x\right)$ if $f_{\bar{P}}\left(x\right)\in C^{0}\left(\bar{\Omega }\right)$ such that it can be approximated by the polynomials to any degree of accuracy [20].

Firstly, we presume that $\bar{M}\leq M$, i.e., β in Equation (4) is an unbiased estimator of $\bar{\beta }$. Hence, the PCE approximation can be considered as a precise approximate of the true function. We compare the performance of the GPCB and the PCE method by comparing their difference in the posterior distribution of the prediction. It is known that given experimental design {X,Y} and kernel function $k\left(x,x^{\prime }\right)=\sum_{l=0}^{\infty }\lambda_{l}\phi_{l}\left(x\right)\phi_{l}\left(x^{\prime }\right)$, the distribution of the prediction of the GPCB reads:

(14) $$\begin{array}{c} p_{G}\left(f_{G}\left(x\right)\right)=N\left(K_{x}^{T}K_{Y}^{-1}Y,K_{\mathrm{xx}}-K_{x}^{T}K_{Y}^{-1}K_{x}\right)≜N\left(\mu_{1},\Sigma_{1}\right), \end{array}$$

where conditions X,Y,x are dropped in $p_{G}\left(f_{G}\left(x\right)|X,Y,x\right)$ for simplicity and $K_{Y}=K+\sigma^{2}I$. Similarly, the prediction of the PCE with the projection method is $f_{P}\left(x\right)=\phi \Phi^{T}WY$, which is derived from the estimation $\beta =\Phi^{T}WY$. Here, $\phi =\phi \left(x\right)\in R^{1\times (P+1)}$, $\Phi =\phi \left(X\right)\in R^{N\times (P+1)}$, $W=diag\{\omega_{1},\ldots ,\omega_{N}\}$ is a diagonal matrix. The corresponding prediction variance is $cov\left(f_{P}\left(x\right)\right)=\phi cov\left(\beta \right)\phi^{T}=\sigma^{2}\phi \Phi^{T}W^{2}\Phi \phi^{T}$. Dropping the conditions in $p_{P}\left(f_{P}\left(x\right)|X,Y,x\right)$ as well, the previous results indicate:

(15) $$\begin{array}{c} p_{P}\left(f_{P}\left(x\right)\right)=N\left(\phi \Phi^{T}WY,\sigma^{2}\phi \Phi^{T}W^{2}\Phi \phi^{T}\right)≜N\left(\mu_{2},\Sigma_{2}\right). \end{array}$$

We can evaluate the discrepancy between $p_{G}\left(f_{G}\left(x\right)\right)$ and $p_{P}\left(f_{P}\left(x\right)\right)$, hence comparing their performance. The KL divergence can be calculated analytically:

(16) $$\begin{array}{c} D_{KL}\left(p_{P},p_{G}\right)=\frac{1}{2}\left(-1+\frac{\Sigma_{2}}{\Sigma_{1}}+\log\frac{\Sigma_{1}}{\Sigma_{2}}+\frac{{\left(\mu_{1}-\mu_{2}\right)}^{2}}{\Sigma_{1}}\right)≜\frac{1}{2}\left(-1+b-\log b+\frac{a^{2}}{\Sigma_{2}}b\right), \end{array}$$

where a,b are simplified notations for the corresponding parts in Equation (16). In fact, *b* is the point-wise ratio between the posterior variances of the predictions, and *a* represents the difference between the posterior mean of the prediction. We discuss the properties of $D_{KL}$ starting from a special case to the general conditions hereafter.

Let *k* be a truncated kernel with the *M* basis of PCE, i.e., $k\left(x,x^{\prime }\right)=\sum_{l=0}^{M}\lambda_{l}\phi_{l}\left(x\right)\phi_{l}\left(x^{\prime }\right)$. Actually, it can be seen as the assignment of $\left\{\lambda_{l},l>M\right\}$ with the value of zero, which can be achieved by optimizing the value $\lambda_{l}$ with a specific procedure. *b* can be simplified as:

(17) $$\begin{array}{cc} b & =\frac{\sigma^{2}\phi \Phi^{T}W^{2}\Phi \phi^{T}}{\phi \left(\Lambda -\Lambda \Phi^{T}K_{Y}^{-1}\Phi \Lambda \right)\phi^{T}}=\frac{\phi \Phi^{T}W^{2}\Phi \phi^{T}}{\phi \Phi^{T}WK_{Y}^{-1}KW\Phi \phi^{T}}\\ & =\frac{\phi \Phi^{T}W^{2}\Phi \phi^{T}}{\phi \Phi^{T}WU{\left(S+\sigma^{2}I\right)}^{-1}U^{T}USU^{T}W\Phi \phi^{T}}\in \left[\frac{s_{\max}+\sigma_{\epsilon }^{2}}{s_{\max}},\frac{s_{\min}+\sigma_{\epsilon }^{2}}{s_{\min}}\right], \end{array}$$

where $\Lambda =diag\{\lambda_{l}\}$ is a diagonal matrix and $K=USU^{T}$ is the eigenvalue decomposition. Let $s_{\max}$ be the maximum eigenvalue and $s_{\min}$ be the minimal one; the above interval holds because *K* is a positive definite matrix. Note that *b* is an invariant with fixed x. On the other hand, the distribution of *a* is as follows:

(18) $$\begin{array}{cc} a & =\phi \left(\Phi^{T}WY-\Lambda \Phi^{T}K_{Y}^{-1}Y\right)=\sigma_{\epsilon }^{2}\phi \Phi^{T}WK_{Y}^{-1}Y\\ & ∼N\left(\sigma_{\epsilon }^{2}\phi \Phi^{T}WK_{Y}^{-1}\hat{Y},\sigma_{\epsilon }^{6}\phi \Phi^{T}WK_{Y}^{-1}K_{Y}^{-1}W\Phi \phi^{T}\right). \end{array}$$

Here, $\hat{Y}$ denotes the mean value of the observations, i.e., the true response. It is necessary to state that the randomness of *a* is brought by the random variable ϵ in observation *Y*. In fact, we have the expectation of $D_{KL}\left(p_{P},p_{G}\right)$ as follows:

(19) $$\begin{array}{cc} E_{\epsilon }\left[D_{KL}\left(p_{P},p_{G}\right)\right] & =\frac{1}{2}\left(-1+b-\log b+\frac{E_{\epsilon }a^{2}}{\Sigma_{2}}b\right)\\ & =\frac{1}{2}\left(-1+b-\log b+\left(var\left(c\right)+{\left(E_{\epsilon }a\right)}^{2}\right)b/\Sigma_{2}\right)\\ & \leq \frac{1}{2}\left(-1+b-\log b+\frac{\sigma_{\epsilon }^{2}+{∥\hat{Y}∥}^{2}}{\sigma_{\epsilon }^{2}}{\left(\frac{\sigma_{\epsilon }^{2}}{s_{\min}+\sigma_{\epsilon }^{2}}\right)}^{2}b\right). \end{array}$$

Presume that the observation *Y* is normalized, as well as $\hat{Y}$; hence, ${∥\hat{Y}∥}^{2}$ can be estimated as O(1). The main difference is affected by the kernel k(·,·) (or Λ) and the term $\sigma_{\epsilon }^{2}$. More specifically, The GPCB can achieve a smaller variance than the PCE method in a point-wise manner because b>1, and the differences between the predictions of the two methods is of the order of $\sigma_{\epsilon }^{2}$. Furthermore, if $\sigma_{\epsilon }^{2}$ is sufficient small, i.e., $\sigma_{\epsilon }^{2}\ll s_{\min}$, we have b→1, thus $E_{\epsilon }\left[D_{KL}\left(p_{P},p_{G}\right)\right]\to 0$. If $\sigma_{\epsilon }^{2}=0$, i.e., we investigate the noise-free models, then b=1 and a=0, which enforces $\Sigma_{1}=\Sigma_{2}$ and $\mu_{1}=\mu_{2}$, respectively. This means that $p_{G}$ and $p_{P}$ are identical distributions, i.e., $D_{KL}\left(p_{P},p_{G}\right)=0$.

We can conclude that the expected value of $D_{KL}\left(p_{P},p_{G}\right)$ is bounded by a certain constant, which mainly depends on the $\sigma_{\epsilon }^{2}$ and Λ. In other words, since $\sigma_{\epsilon }^{2}$ is given in the prior, and the Λ are optimized; hence, the $D_{KL}\left(p_{P},p_{G}\right)$ is constrained, which means that the GPCB is as stable as the PCE method. Nonetheless, if $f_{P}$ has reached a desired prediction precision, then $f_{G}$ with the kernel constructed with the same basis can have a desirable precision, as well as smaller variance.

Secondly, we consider that $\bar{M}>M$, where the β of the PCE method is not an unbiased estimate of $\bar{\beta }$ any longer. Let $p_{\bar{P}}$ denote the PCE approximation with the $\bar{M}$ basis; under the circumstance, $k\left(x,x^{\prime }\right)=\sum_{k=0}^{\bar{M}}\lambda_{l}\phi_{l}\left(x\right)\phi_{l}\left(x^{\prime }\right)$ is achieved by tuning the value of Λ via a certain learning method; hence, $D_{KL}\left(p_{\bar{P}},p_{G}\right)$ is also bounded by a constant according to Equation (19), i.e., the GPCB can converge to the precise PCE prediction $p_{\bar{P}}$, as well. However, the KL divergence $D_{KL}\left(p_{\bar{P}},p_{P}\right)$ is given as:

(20) $$\begin{array}{c} \begin{array}{cc} D_{KL}\left(p_{\bar{P}},p_{P}\right) & =\frac{1}{2}\left(-1+\frac{\bar{\Sigma }}{\Sigma_{2}}-\log\frac{\bar{\Sigma }}{\Sigma_{2}}+\frac{{\left(\bar{\mu }-\mu_{2}\right)}^{2}}{\Sigma_{1}}\right)≜\frac{1}{2}\left(-1+\bar{b}-\log\bar{b}+\frac{{\bar{a}}^{2}}{\Sigma_{1}}\right),\\ \bar{b} & =\frac{\phi \Phi^{T}W^{2}\Phi \phi^{T}+\phi_{r}\Phi_{r}^{T}W^{2}\Phi_{r}\phi_{r}^{T}+\phi A\phi_{r}^{T}}{\phi \Phi^{T}W^{2}\Phi \phi^{T}}\geq 1,\\ \bar{a} & =\phi_{r}\Phi_{r}^{T}WY∼N\left(\phi_{r}\Phi_{r}^{T}W\hat{Y},\sigma_{\epsilon }^{2}\phi_{r}\Phi_{r}^{T}W^{2}\Phi_{r}\phi_{r}^{T}\right). \end{array} \end{array}$$

We denote $\phi_{r}$ as the basis that belongs to the model $f_{\bar{P}}$, but $f_{P}$. It is shown that the biased PCE $f_{P}$ has smaller variance, however with a bias whose mean value depends on $\phi_{r}$. We notice that $\phi_{r}$ represents the high-order polynomials; hence, the bias can be considerably large for general cases, and so is the $D_{KL}\left(p_{\bar{P}},p_{P}\right)$. We can conclude that even though the biased PCE has smaller variance, the relatively large bias can lead to a false prediction.

In fact, if the underlying system is smooth enough to be modeled by a polynomial approximation, then we can adaptively increase the number of polynomial bases (and the experimental designs if necessary) to reach a precise approximation. However, on the other hand, we can directly use the GPCB method, which is a one-step Bayesian approximation, that converges to the hypothetical true system $f_{\bar{P}}$. Roughly speaking, the GPCB finds $\bar{M}$ automatically by tuning the parameters Λ instead of adaptively changing the value of *P* in the PCE method. It indeed provides more convenience for computation. The key problem is the evaluation of Λ. Specifically, we introduce the Mehler kernel [37], which is an analytic expression of the Mercer kernel constructed by Hermite polynomials, and discuss the learning procedure of $\Lambda_{l}$.

### 4.2. Construction of the Kernel with Hermite Polynomials

Recall that we have $\left\{\phi_{\alpha }\right\}$ in PCE as an orthonormal basis, then we regard them as eigenfunctions of a kernel k(·,·). Since p(x) follows the standard Gaussian distribution, then $\phi_{l}^{\left(i\right)}\left(x_{i}\right)=He_{l}\left(x_{i}\right)/\sqrt{l!}$, where $x_{i}$ is the *i*-th variable of x and $He_{l}\left(x_{i}\right)$ is the Hermite polynomial of degree *l*:

(21) $$\begin{array}{c} He_{l}\left(x_{i}\right)={\left(-1\right)}^{l}e^{\frac{x_{i}^{2}}{2}}\frac{d^{l}}{dx_{i}^{l}}e^{-\frac{x_{i}^{2}}{2}}. \end{array}$$

**Figure 2:**
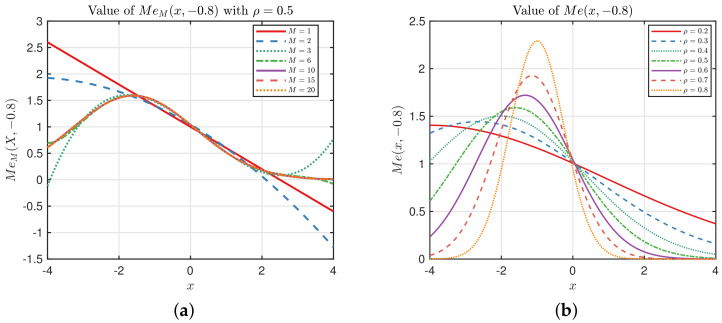
Comparison of the effect of *M* and ρ on the 1D Mehler kernel with one fixed point {−0.8}. (**a**) Kernel value of $Me_{M}\left(x,-0.8\right)$ with ρ=0.5; (**b**) kernel value of Me(x,−0.8).

Here, we denote the real *l*-th multi-index of the *l*-th polynomial in Equation (2) as $\alpha^{\left(l\right)}=\left(\alpha_{1}^{\left(l\right)},\ldots ,\alpha_{d}^{\left(l\right)}\right)$, $|\alpha^{\left(l\right)}|=\alpha_{1}^{\left(l\right)}+\ldots +\alpha_{d}^{\left(l\right)}$ and $\alpha^{\left(l\right)}!=\alpha_{1}^{\left(l\right)}!\ldots \alpha_{d}^{\left(l\right)}!$, then according to the previous analysis, we can get $\phi_{\alpha^{\left(l\right)}}\left(x\right)={\mathrm{He}}_{\alpha^{\left(l\right)}}\left(x\right)/\sqrt{\alpha^{\left(l\right)}!}$. We have Mehler kernel $Me(X,X^{\prime })$ [37] with orthonormal Hermite polynomials as eigenfunctions:

(22) $$\begin{array}{c} Me\left(x,x^{\prime }\right)=\exp\left\{-\frac{D_{x}D_{x}^{T}+D_{x^{\prime }}D_{x^{\prime }}^{T}}{2}\right\}\left[\exp\sum_{i=1}^{d}\rho_{i}x_{i}x_{i}^{\prime }\right]=\sum_{l=0}^{\infty }\rho^{\alpha^{\left(l\right)}}\phi_{\alpha^{\left(l\right)}}\left(x\right)\phi_{\alpha^{\left(l\right)}}\left(x^{\prime }\right), \end{array}$$

where the eigenvalue is $\lambda_{\alpha^{\left(l\right)}}=\rho^{\alpha^{\left(l\right)}}=\prod_{i=1}^{d}\rho_{i}^{\alpha_{i}^{\left(l\right)}}$ for parameter ρ, $D_{x}$ is the symbol representing the row gradient operator, i.e., $D_{x}=\left(\partial /\partial x_{1},\ldots ,\partial /\partial x_{d}\right)$, and $D_{x^{\prime }}$ is defined similarly. Specifically, in the one-dimensional case:

(23) $$\begin{array}{c} Me\left(x,x^{\prime }\right)=\frac{1}{\sqrt{1-\rho^{2}}}\exp\left(-\frac{\rho^{2}\left(x^{2}+{x\prime }^{2}\right)-2\rho xx^{\prime }}{2(1-\rho^{2})}\right)=\sum_{l=0}^{\infty }\rho^{l}\phi_{l}\left(x\right)\phi_{l}\left(x^{\prime }\right), \end{array}$$

where the eigenvalue $\lambda_{l}=\rho^{l}>0$. The truncated kernel $Me_{M}\left(x,x^{\prime }\right)=\sum_{l=0}^{M}\rho^{l}\phi_{l}\left(x\right)\phi_{l}\left(x^{\prime }\right)$, and its attribution can be investigated by varying ρ and *M*. Figure 2a illustrates the truncated kernel $Me_{M}\left(x,x^{\prime }\right)$, which shows that $Me_{M}\left(x,x^{\prime }\right)$ tends to converge to $Me(x,x^{\prime })$ as *M* grows. Figure 2b shows the values of Me(x,−0.8) with different ρ. It presents to us that the influence of eigenvalue $\lambda_{l}$ is greater on the Mehler kernel.

### 4.3. Learning the Hyper-Parameter ρ of $Me(x,x^{\prime })$

**Figure 3:**
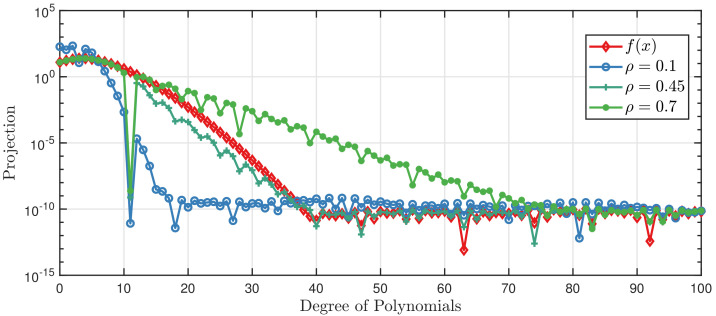
Projections on the first 100 polynomials of f(x) and the Gaussian process on polynomial chaos basis (GPCB) with ρ=0.1,0.45,0.7.

It is clear that $\lambda_{l}=\rho^{l}$ has a great impact on the kernel values, hence affecting the convergence of the $f_{G}\left(x\right)$. We start with a simple example, where $f\left(x\right)=5+x+\exp\left(x\right),x∼N\left(0,2^{2}\right)$ is the true underlying function, and the noise term is ignored in the observation. Considering the Taylor expansion of exp(x), f(x) can be approximated by the PCE with sufficiently large *M*. In fact, we can calculate the projection $<f\left(x\right),\phi_{l}\left(x\right)>$ to seek the value of *M*. When l>M, $<f\left(x\right),\phi_{l}\left(x\right)>\approx 0$. On the other hand, as discussed in Section 4.1, the GPCB can find *M* automatically by tuning the hyper-parameter ρ. Let the experimental design X be the zeros of $He_{10}\left(x\right)$ of Equation (21), i.e., quadrature points corresponding to degree 10; we compare the performance of the GPCB with ρ equal to 0.1,0.45,0.7, respectively. The results are displayed in Figure 3. Note that the projection value is the absolute value of the true value in the figure for better illustration. It shows that we are able to approximate f(x) with polynomials up to degree 40. If ρ=0.45 for the Mehler kernel, the GPCB almost converges to exact f(x), whereas ρ=0.1 leads to a fast convergence rate and ρ=0.7 results in a slow convergence rate.

Figure 3 shows that the ρ has a crucial impact on the performance of the GPCB method, so a tractable method to optimize ρ is needed. A natural criterion is the KL divergence, which can be minimized by finding the optimal hyper-parameters ρ. We discussed the KL divergence of the GPCB and the PCE surrogates under the assumption that the PCE surrogate model can approximate the true system to any degree of accuracy. However, the distribution of the real system is usually unknown, which makes the calculation of KL divergence intractable. In fact, it can be easily deduced that the minimization of KL divergence is equivalent to minimizing the negative log marginal likelihood Δ, which (actually is 2Δ) reads:

(24) $$\begin{array}{c} \Delta =Y^{T}K_{Y}^{-1}Y+\log\left[{\left(2\pi \right)}^{N}\left|K_{Y}\right|\right] \end{array}$$

It is important to optimize ρ and $\sigma_{\epsilon }^{2}$ to obtain a suitable kernel to get an accurate approximation. Classical methods like gradient-based techniques can be used to search for the optimal ρ; however, it may perform poorly because it is locally optimized. As we can see in Equation (23), it is indicated that ρ should take a value between zero and one, so we can propose a global method to solve our optimization problem. The algorithm for generating a GPCB approximation is given in Algorithm 1: 
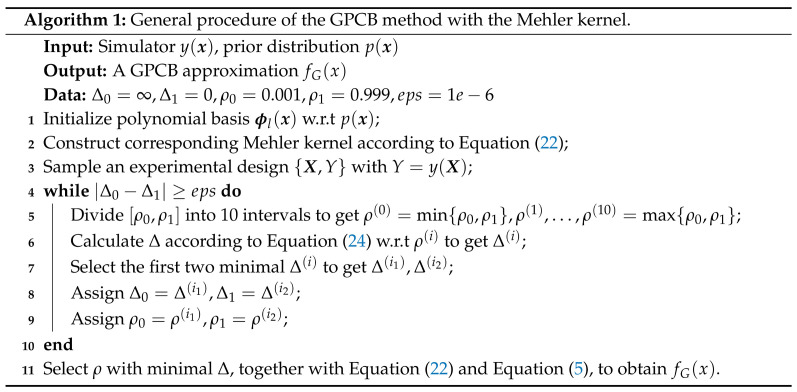


## 5. Numerical Investigation

**Figure 4:**
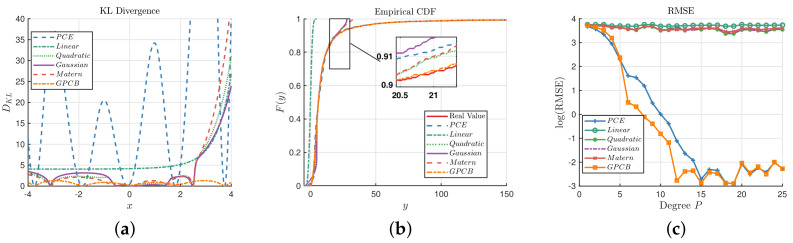
Comparisons among the GP, polynomial chaos expansion (PCE) and GPCB surrogates for the 1D example. (**a**) Comparison of the KL divergence, P=10; (**b**) comparison of the ECDF of prediction, P=10; (**c**) comparison of the RMSE with different degrees *P* in the 1D example.

In this section, we investigate the GPCB method for various benchmark functions. Firstly, we investigate the same example in Figure 3; however, the noise term is considered, i.e., y=f(x)+ϵ=5+x+exp(x)+ϵ is the observation, where $x∼N\left(0,2^{2}\right),\epsilon ∼N\left(0,0.1^{2}\right)$. Three methods, i.e., GP with the RBF kernel, PCE and GPCB, are implemented. It is necessary to note that the Monte Carlo (MC) sampling strategy is used in the normal GP approaches, and Gaussian quadrature points are introduced in the GPCB approach. The main reason is that the quadrature points are too sparse for the widely-used kernels to capture the local features. For example, in this case, P=10 in PCE, then the maximum quadrature point is 10.376, which is beyond $3\sigma_{x}$. In the first set of experiments, let P=10 in PCE, which means 11 sample points are used in the experiments; furthermore, 10,000 samples are introduced as test dataset to output the ECDF (empirical cumulative distribution function) and RMSE (root-mean-squared error). The GP algorithm is implemented by the gpml toolbox [38] written in MATLAB with four different kernels, i.e., linear, quadratic, Gaussian and Matérn-3/2 kernels. The comparisons of the results are displayed in Figure 4.

Figure 4a illustrates the point-wise KL divergence between the true value of f(x) and the predictions on the interval [−4,4] based on Equation (16). It is clear that the distribution of GPCB prediction is statistically closest to the true response, although the GP methods with quadratic, Gaussian and Matérn-3/2 kernels outperform the GPCB at some points. Figure 4b compares the ECDF of *y* based on the test dataset. It shows that both the PCE and GPCB have a similar ECDF with the true value. Upon closer inspection, which is shown in the magnified subregion, it is obvious that the ECDF of the GPCB is almost exactly the same as the real ECDF, which shows that GPCB has actually captured the feature of f(x) with high precision. At the same time, the RMSEs of the PCE, linear kernel, quadratic kernel, Gaussian kernel, Matérn-3/2 kernel and GPCB are 1.0570,37.3526,23.6383,25.6044,27.4489,0.4108, respectively. We have implemented another experiment, which uses the degree *P* (i.e., the number of experimental design) as the second set of experiments, which are illustrated in Figure 4c. Figure 4c shows that GPCB generally outperforms the ordinary GP with the RBF kernel, which indicates that GPCB performs better with a few (or sparse) training points. It is notable that the PCE and GPCB perform with almost the same precision when the degree is greater than 16. It echoes the idea that PCE and GPCB are statistical equivalent, as we present in Section 4.1.

**Figure 5:**
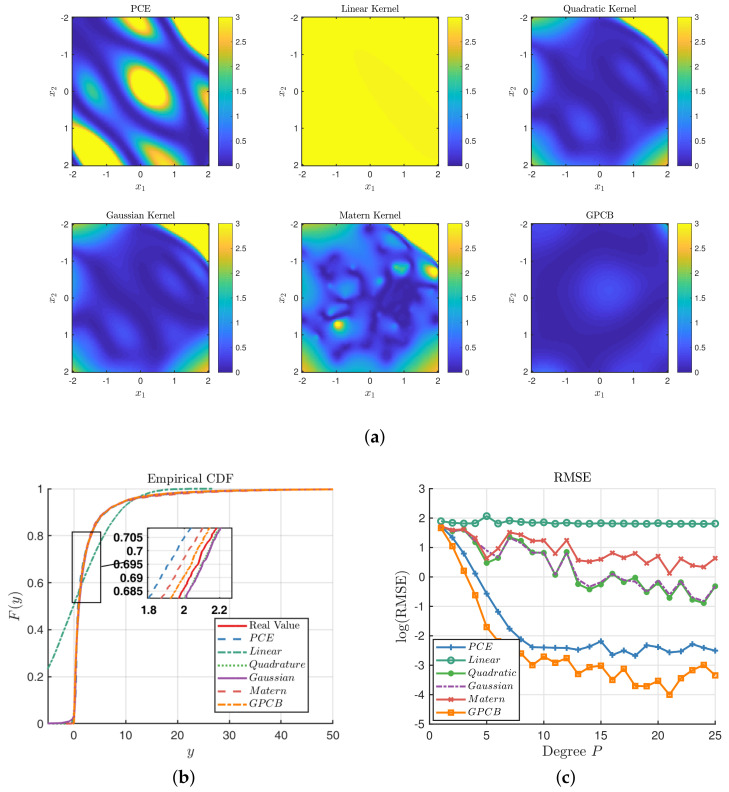
Comparisons among the GP, PCE and GPCB surrogates for the 2D example. (**a**) Comparison of the KL divergence with $p_{t}=7$; (**b**) comparison of the ECDF of prediction, $p_{t}=7$ ; (**c**) comparison of the RMSE with different degrees $p_{t}$ in the 2D example.

Similar experiments are conducted with a two-dimensional function, which is expressed as $f\left(x\right)=\exp\left(x_{1}\right)/\exp\left(x_{2}\right)$. Let $x_{1},x_{2}∼N\left(0,1\right)$, y=f(x)+ϵ be the real model where ϵ is an independent noise term with a normal distribution $N(0,0.1^{2}I_{2})$. Unlike the first test function, this test example is a limit state function. Let the maximum degree for each dimension $p_{t}$ be seven for the PCE method, which makes 64 training points in total. Another dataset of 10,000 independent samples is introduced as the test set to calculate the ECDF and RMSE, as well. Similarly, we have the point-wise KL divergence in the region [−2,2;−2,2] as shown in Figure 5a. It is clear that the GPCB is globally closer to the true distribution than other methods. The GP with quadratic, Gaussian and Matérn-3/2 kernels can approximate the center part well, while the PCE does not seem to perform as well. Figure 5b shows that the six methods except the linear kernel are able to reconstruct the distribution of the prediction, and upon closer observation, we find that the ECDF of the GPCB and Matérn-3/2 kernel are the best approximations among the six methods. We also consider another set of experiments focusing on the number of experimental designs, which equals ${\left(p_{t}+1\right)}^{2}$ for the 2D function. The RMSEs of the three methods with respect to different $p_{t}$ are displayed in Figure 5c. It also shows that the PCE and GPCB generally outperform the normal GP approaches, and the GPCB has the best performance.

To summarize, the GPCB generates a surrogate of infinite series, while the PCE can only generate a surrogate with up to P+1 polynomials, and they tend to behave with similar precision when *P* is large enough. A set of sparse quadrature points sampled in the PCE, which are derived from the Gaussian quadrature rule, is a good design for the GPCB. The GPCB with those training points generally performs better than the normal GP methods and PCE. However, the size of such a training set grows dramatically with the dimension ($N={\left(p_{t}+1\right)}^{d}$ in total), so it is not practical in real-life applications. We aim to present a strategy of sampling from those quadrature points, namely candidate points in the next section, and analyze the performance of our algorithm on the selected points.

### 5.1. A Random Constructive Design in High Dimensional Problems

As the dimension of a system grows, so do the number of design points of PCE due to a tensor product of quadrature points in each dimension. It is possible that PCE could deal with thousands of points of training data with acceptable computational time; however, it becomes expensive for GP approaches, including our GPCB approach. Monte Carlo sampling techniques can substitute quadrature design; however, these are not always stable. Other sampling strategies like Halton sampling and Latin hypercube sampling [39] are widely used.

In this work, we want to utilize the high accuracy of quadrature points and also want to reduce the massive number of points. Let $x\in R^{d}$, and $p_{t}$ is the maximum degree in each dimension, so $p_{t}+1$ quadrature points are needed in each dimension, which makes the total number of tensor products of quadrature points be $\#\left\{X_{c}\right\}={\left(p_{t}+1\right)}^{d}$. We seek to find a subset of the candidate design $X_{c}$. Furthermore, we wish to obtain a subset having a good coverage rate in the space. Therefore, we proposed the random definite design in our paper. Note that the LHS design can be extended to a larger interval $\left(1,{\left(p_{t}+1\right)}^{D}\right)$ and can produce points at midpoints (endpoints), so we use the LHS design to sample *N* indices from the interval. More specifically, we presume that the points in $X_{c}$ are equally important, so we arrange those points with a certain order to get their indices. Then, we sample from the indices with the LHS design, and each index is related to a certain quadrature point. It can be easily implemented by the MATLAB built-in function lhsdesign. The corresponding *N* points are what we need.

**Figure 6:**
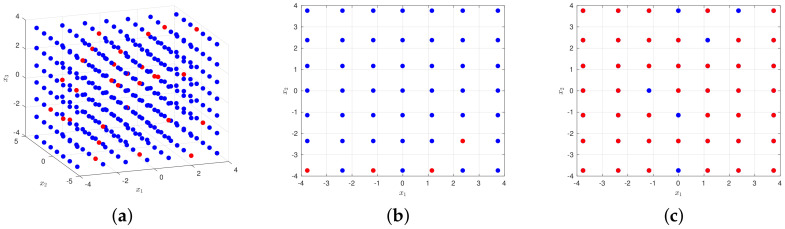
Left: $X_{c}$ and X in 3D view; the blue dots represent the quadrature points, while the red points represent our samplings; middle: this shows the sparsity of our sampling in $X_{c}$; right: this shows that our sampling actually covered almost every feature of $X_{c}$. (**a**) $X_{c}$ and X in 3D view; (**b**) one slice of $X_{c}$; (**c**) projection of X on $X_{c}$ in dimensions one and two.

Take a three-dimensional input space as an example, where $x_{i}∼N\left(0,1\right),i=1,2,3$. Set $p_{t}=6$, then $\#\{X_{c}\}=343$. The candidate design and its subset of 50 points X are illustrated in Figure 6. We can see from the figure that our sampling is sparse in the whole set of candidate points, and it behaves uniformly in dimension one as illustrated in Figure 6b, with similar conclusions in the other two dimensions. When projecting our sampling from dimension three to get Figure 6c, we can see that the selected points almost cover every point of $X_{c}$, which means it has all features in dimensions one and two, i.e., quadrature point values of the two dimensions. It shows that such a method can generate a sparse subset meanwhile guaranteeing the coverage rate in the whole candidate design. We name it the random constructive design.

**Figure 7:**
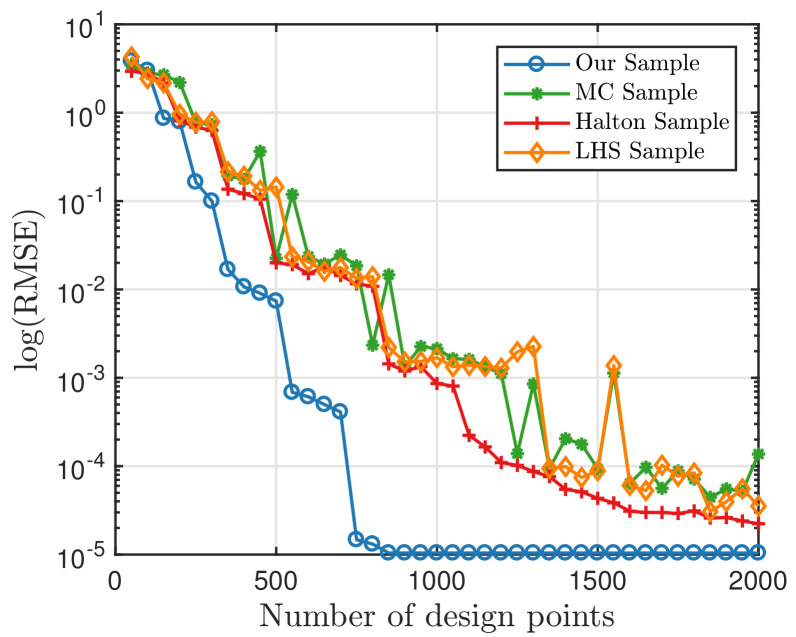
Comparison of the RMSE between four sampling strategies with the PCE method.

Now, we want to find out whether these samples retain their capability of accuracy. Firstly, we use the PCE method to test those samples. We will look into the benchmark Ishigami function [40]: $f\left(x\right)=\sin\left(x_{1}\right)+7\sin^{2}\left(x_{2}\right)+0.1x_{3}^{4}\sin\left(x_{1}\right)$, where four different sampling strategies are compared here. Set $p_{t}=15$, and the candidate design $X_{c}$ has a size of 4096. The error term ϵ is eliminated in this simulation for the accuracy test. The RMSE are computed on 10,000 independently-sampled data, and the results are presented below in Figure 7. It can be seen that our sample always performs better than other samples. When the number of samples surpasses 900, the RMSE becomes $1.0605\times 10^{-5}$, which equals the RMSE with the whole candidate points. Therefore, we only select 20% of $X_{c}$ and get the same precision. Furthermore, if we set our precision to be $10^{-2}$, only 400 points are needed. This shows that the quadrature points have high precision in numerical calculation. In other words, the points in the candidate set are good points.

**Figure 8:**
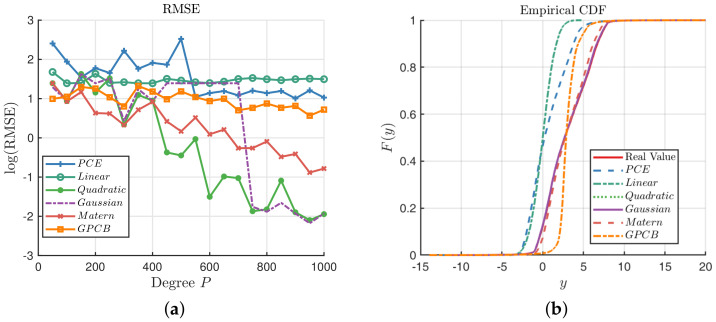
Comparisons among the GP, PCE and GPCB surrogates for the Ishigami function. (**a**) Comparison of the RMSE; (**b**) comparison of the ECDF; N=1000.

Then, the random constructive design is used with the PCE, GP and GPCB methods for the Ishigami function, with the noise term added in the observations. We take the RMSE as a criterion to compare their performance, and the results are illustrated in Figure 8. Figure 8a shows that the GPCB is always better than the PCE method, and they tend to behave the same. However, as the number of sampling points grows, the GP with the quadratic kernel, Gaussian kernel and Matérn-3/2 kernel generally outperform the other methods. We can see that the Ishigami function is a bounded function; therefore, it is likely to fill the whole observation space as the number of samples increases, hence improving the accuracy of the GP method. We plot the ECDF with respect to the three methods when N=1000 in Figure 8b. It is clear that the GP with the quadratic kernel, Gaussian kernel and Matérn-3/2 kernel can almost recover the true distribution of the response, which is beyond the capability of the PCE and GPCB.

A six-dimensional problem is being tested with the G-function [41], which is not like the Ishigami function and is unbounded in the domain ${\left[-\infty ,\infty \right]}^{6}$:

(25) $$\begin{array}{c} f\left(x\right)=\prod_{i=1}^{6}\frac{|4x_{i}-2|+a_{i}}{1+a_{i}},\;where\;a_{i}=\frac{i-2}{2},\;\forall i=1,\ldots ,6 \end{array}$$

**Figure 9:**
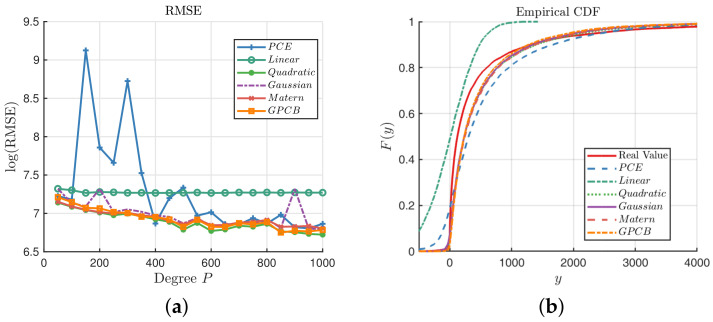
Comparisons among the GP, PCE and GPCB surrogates for the G-function. (**a**) Comparison of the RMSE; (**b**) comparison of the ECDF; N=1000.

The experiment is performed with the same approaches, and the results are shown below in Figure 9. Figure 9a shows that the GPCB outperforms the PCE and GP with the Gaussian Kernel, and it has similar precision with the quadratic and Matérn-3/2 kernels. We notice that the GPCB is more stable than the PCE method, which behaves badly especially when N=150,300. Figure 9b shows that none of these three methods can reconstruct the probability of *y* very well; however, we can note that the GPCB is still comparatively the closest.

Finally, we are going to present a more complicated model with 15 dimensional functions with the following form:

(26) $$\begin{array}{c} f\left(x\right)=a_{1}^{T}x+a_{2}^{T}\sin\left(x\right)+a_{3}^{T}\cos\left(x\right)+x^{T}Mx. \end{array}$$

**Figure 10:**
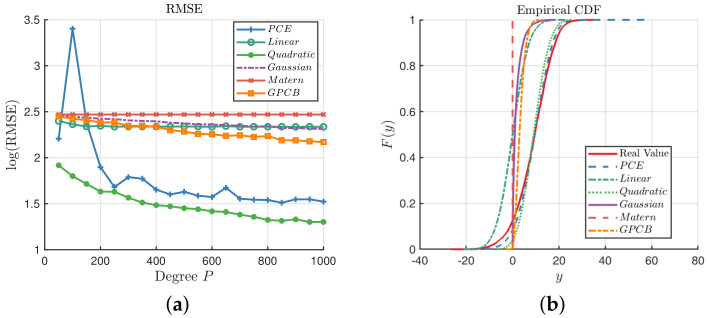
Comparisons among the GP, PCE and GPCB surrogates for Equation (26). (**a**) Comparison of the RMSE; (**b**) comparison of the ECDF; N=1000.

The distribution of x is the product of 15 independent distributions, i.e., $x_{i}∼N\left(0,1\right),i=1,\ldots ,15$. This function is introduced by the work of O’Hagan, where $a_{1},a_{2},a_{3},M$ are defined in [42]. We can see that this function is dominated by the linear and quadratic term, so it may be well approximated by the low-order PCE model. Let $p_{t}=3$ in the PCE model; we can see from Figure 10a that the GP with the quadratic kernel performs best among the six methods, while the PCE performs better than the GPCB and other GP methods. On the other hand, the GPCB is always generally better than the GP method except with the quadratic kernel for this function. When N=1000, the PCE can generate *y*, which follows the real distribution according to the ECDF in Figure 10b.

## 6. Conclusions

This paper has examined two different surrogates of computational models, i.e., polynomial chaos expansion and Gaussian process regression. First, we present a brief review of these two approaches. Next, we discuss the relationship between PCE and GP and find that PCE and GP surrogates are embedded in two isomorphic RKHS. Mercer’s theorem is introduced to generate a kernel based on a PCE basis, by which a new approach is proposed, which we name GPCB. An example shows that with the same experimental design, GPCB tends to retain useful information in a suitable subspace of the RKHS by changing the hyper-parameters, whereas PCE simply sets the information of the residual to zero. We further investigate the approximation performance on two test functions in 1D and 3D, respectively, and their approximation properties are illustrated. In order to deal with the high dimensional scenario, a random constructive design from the quadrature points is used to generate an experimental design. The results give us several directions for choosing models: basically, the GPCB outperforms the PCE, but when the original model can be well approximated by low-order PCE (Figure 10), it seems cumbersome to introduce the GPCB and GP; when the response function is bounded (Figure 8), if we have enough training resources, the GP can be a better choice; when the objective function is unbounded (Figure 4 and Figure 9) or cannot be approximated by finite polynomials (Figure 5), we should probably choose the GPCB.

Future work can extend the family of the Mercer kernel or equivalent kernel (other than the Mehler kernel presented in this paper) beyond a classical approximation method. We can also analyze the experimental design for GP regression in many ways. Although we can see that our sampling method behaves fair enough in the experiments, there is also the opportunity to discover further suitable experimental design schemes to fit different computational purposes, which would be of great interest. The stability of our method will be investigated in future work, i.e., how many points are needed to train a good surrogate and whether our method always produces a suitable design. Furthermore, we can establish closer connections between numerical analysis and statistics via such combinations.

## Appendix A. Proofs of Proposition 1, Proposition 3, Proposition 2 and Theorem 1

Here, we use the same notations as in Section 3.

### Appendix A.1. Proof of Proposition 1

**Proof.** Define the inner product in the above subspace $H_{P}$ as follows:

(A1) $$\begin{array}{c} <f\left(x\right),g\left(x\right){>}_{H_{P}}=\sum_{l}f_{l}g_{l}/\lambda_{l}. \end{array}$$

Firstly, it is obvious that $H_{P}$ is a Hilbert space in H. Secondly, for any x∈Ω, k(x,·) belongs to $H_{P}$ because:

(A2) $$\begin{array}{c} <k\left(x,\cdot \right),k\left(x,\cdot \right){>}_{H_{P}}=\sum_{l}\frac{<k\left(x,\cdot \right),\phi_{l}\left(\cdot \right){>}^{2}}{\lambda_{l}}=\sum_{i}\lambda_{l}\phi_{l}^{2}\left(x\right)<\infty . \end{array}$$

It also has the reproducing property for:

(A3) $$\begin{array}{c} <f\left(\cdot \right),k\left(x,\cdot \right){>}_{H_{P}}=\sum_{l}f_{l}\lambda_{l}\phi_{l}\left(x\right)/\lambda_{l}=f\left(x\right)\;for\;\forall \;x\in \Omega , \end{array}$$

We have the conclusion that $H_{P}$ is an RKHS derived from the Mercer kernel. □

### Appendix A.2. Proof of Proposition 2

**Proof.** In fact, $H_{G}^{\prime }$ is a space of all finite linear combinations of functions k(x,·):Ω→R, so we can denote $H_{G}^{\prime }:=span\left\{k\left(x,\cdot \right)|x\in \Omega \right\}$, and the elements in $H_{G}^{\prime }$ have the general form of $f\left(x\right)=\sum_{i=1}^{N}f_{i}k\left(x,X_{i}\right)$. Therefore, different *N* and all experimental designs X are allowed, which enables that $f\left(x\right)=\sum_{i=1}^{N}f_{i}k\left(x,X_{i}\right),g\left(x\right)=\sum_{i=1}^{N^{\prime }}f_{i}k\left(x,X_{i}^{\prime }\right)\in H{‘}_{G}$. The linearity of $H_{G}^{\prime }$ is given by the following explanation. Let $t_{1},t_{2}\in R$ be scalars, then we can rewrite $t_{1}f^{\left(1\right)}\left(x\right)+t_{2}f^{\left(2\right)}\left(x\right)$ as a function f(x) such that:

(A4) $$\begin{array}{c} f\left(x\right)=\sum_{i=1}^{N_{1}}t_{1}f_{i}^{\left(1\right)}k\left(x,X_{i}^{\left(1\right)}\right)+\sum_{j=1}^{N_{2}}t_{2}f_{j}^{\left(2\right)}k\left(x,X_{j}^{\left(2\right)}\right)≜\sum_{l=1}^{N}f_{l}k\left(x,X_{l}\right), \end{array}$$

where $N=N_{1}+N_{2}$, $\left\{f_{l},l=1,\ldots ,N\right\}=\left\{t_{1}f_{i}^{\left(1\right)},i=1,\ldots ,N_{1}\right\}\cup \left\{t_{2}f_{j}^{\left(2\right)},j=1,\ldots ,N_{2}\right\}$, $X\;=\;X^{\left(1\right)}\cup \;X^{\left(2\right)}$. Additionally, we have:

(A5) $$\begin{array}{cc} \sum_{l=1}^{N}\sum_{m=1}^{N}f_{l}f_{m}k\left(X_{l},X_{m}\right)= & \sum_{i=1}^{N_{1}}\sum_{i^{\prime }=1}^{N_{1}}t_{1}^{2}f_{i}^{\left(1\right)}f_{i^{\prime }}^{\left(1\right)}k\left(X_{i}^{\left(1\right)},X_{i^{\prime }}^{\left(1\right)}\right)\\ & +\sum_{j=1}^{N_{2}}\sum_{j^{\prime }=1}^{N_{2}}t_{2}^{2}f_{j}^{\left(2\right)}f_{j^{\prime }}^{\left(2\right)}k\left(X_{j}^{\left(2\right)},X_{j^{\prime }}^{\left(2\right)}\right)+2\sum_{i=1}^{N_{1}}\sum_{j=1}^{N_{2}}t_{1}t_{2}f_{i}^{\left(1\right)}f_{j}^{\left(2\right)}k\left(X_{i}^{\left(1\right)},X_{j}^{\left(2\right)}\right)\\ & <+\infty , \end{array}$$

which means that $t_{1}f^{\left(1\right)}\left(x\right)+t_{2}f^{\left(2\right)}\left(x\right)$ also belongs to $H_{G}^{\prime }$. Then, we would like to show that $H_{G}^{\prime }$ is an inner product space. Let the kernel function be positive semi-definite, and we define the inner product of $H_{G}^{\prime }$ as follows:

(A6) $$<f\left(x\right),g\left(x\right){>}_{H_{G}^{\prime }}=\sum_{i=1}^{N}\sum_{j=1}^{N^{\prime }}f_{i}g_{j}k\left(X_{i},X_{j}^{\prime }\right).$$

$<\cdot ,\cdot {>}_{H_{G}^{\prime }}$ is a well-defined inner product by checking the following conditions:

1. Symmetry: $<f,g{>}_{H_{G}^{\prime }}=\sum_{i,j}f_{i}g_{j}k\left(X_{i},X_{j}^{\prime }\right)=\sum_{j,i}g_{j}f_{i}k\left(X_{j}^{\prime },X_{i}\right)=<g,f{>}_{H_{G}^{\prime }}$;
2. Bi-linearity:
  $$\begin{array}{cc} <t_{1}f^{\left(1\right)}+t_{2}f^{\left(2\right)},g{>}_{H_{G}^{\prime }}= & \sum_{l=1}^{N}\sum_{j=1}^{N^{\prime }}f_{l}g_{j}k\left(X_{l},X_{j}^{\prime }\right)\\ = & a\sum_{i=1}^{N_{1}}\sum_{j=1}^{N^{\prime }}f_{i}^{\left(1\right)}g_{j}k\left(X_{i}^{\left(1\right)},X_{j}^{\prime }\right)+b\sum_{i=1}^{N_{2}}\sum_{j=1}^{N^{\prime }}f_{i}^{\left(2\right)}g_{j}k\left(X_{i}^{\left(2\right)},X_{j}^{\prime }\right)\\ = & a<f^{\left(1\right)},g{>}_{H_{G}^{\prime }}+b<f^{\left(2\right)},g{>}_{H_{G}^{\prime }}; \end{array}$$
3. Positive-definiteness: It is obvious that $<f,f{>}_{H_{G}^{\prime }}=f^{T}Kf\geq 0$ with the equality iff f=0.

 □

### Appendix A.3. Proofs of Proposition 3

**Proof.** Firstly, we can prove that the reproducing formula holds for the space $H_{G}^{\prime }$. For any *X*, k(X,x) is a function of x and belongs to $H_{G}^{\prime }$. Furthermore, we have:

(A7) $$\begin{array}{c} <f\left(\cdot \right),k\left(x,\cdot \right){>}_{H_{G}^{\prime }}=\sum_{i=1}^{N}f_{i}k\left(x,X_{i}\right)=f\left(x\right). \end{array}$$

The above reproducing property is valid for any $f\in H_{G}^{\prime }$; thus, it is still valid for the closure $H_{G}$ in the sense of generalizing the above equation as $<f\left(\cdot \right),k\left(x,\cdot \right){>}_{H_{G}}=f\left(x\right)$. Then, we need to prove that $H_{G}$ is unique. Suppose that we have another Hilbert space $H_{G^{\prime }}$, which is possibly an RKHS of the kernel k(·,·), then, for a specific X, we can get:

(A8) $$\begin{array}{c} <k\left(x,X\right),k\left(x^{\prime },X\right){>}_{H_{G}^{\prime }}=k\left(x,x^{\prime }\right)=<k\left(x,X\right),k\left(x^{\prime },X\right){>}_{H_{G^{\prime }}}. \end{array}$$

This proves that the two inner products are the same on $H_{G}^{\prime }$; then, $H_{G^{\prime }}$ must contain $H_{G}$ because it is the closure of $H_{G}^{\prime }$. $H_{G^{\prime }}$ must be equivalent to $H_{G}$, otherwise we can find a nonzero element $f\in H_{G^{\prime }}-H_{G}$ such that it is orthogonal to $H_{G}$. However, we can always get that $f=<f,k\left(\cdot ,X\right){>}_{H_{G^{\prime }}}\equiv 0$ for a particular X, which is a contradiction. □

### Appendix A.4. Proofs of Theorem 1

**Proof.** Define a weighted $l^{2}$ space such that:

(A9) $$\begin{array}{c} l_{\lambda }^{2}=\left\{h|<h,h{>}_{l_{\lambda }^{2}}=\sum_{l}\lambda_{l}h_{l}^{2}<\infty \right\}. \end{array}$$

It is clear that $\left\{c_{l}\left(X\right)\right\}\in l_{1/\lambda }^{2}$ for $c_{l}\left(X\right)$ defined in Equation (12). $l_{1/\lambda }^{2}$ is the completion of the span of all $\left\{c_{i}\left(X\right)\right\}$, then $H_{G}$ is isometrically isomorphic to $l_{1/\lambda }^{2}$, because firstly, $c_{l}\left(X\right)$ is identically determined by k(·,·) and X, secondly, according to Equation (13), $<f_{X}\left(x\right),g_{X^{\prime }}\left(x\right){>}_{H_{G}}=\sum_{l}c_{l}\left(X\right)c_{l}\left(X^{\prime }\right)/\lambda_{l}$. On the other hand, there exists a linear map such that:

(A10) $$\begin{array}{c} T:l_{1/\lambda }^{2}\to H_{P},\;T\left(c\right)=\sum_{l}c_{l}\phi_{l}. \end{array}$$

This is a surjective map for every $f\in H_{P}$, $\left\{f_{i}\right\}\in l_{1/\lambda }^{2}$. Now, we need to prove that the projection *T* is injective. Assume there exist *c* and $c^{\prime }$ such that $T\left(c\right)=T\left(c^{\prime }\right)$, then:

(A11) $$\begin{array}{c} 0=∥T\left(c\right)-T\left(c^{\prime }\right){∥}_{H_{P}}^{2}=<T\left(c-c^{\prime }\right),T\left(c-c^{\prime }\right){>}_{H_{P}}=\sum_{l}{\left(c_{i}-c_{i}^{\prime }\right)}^{2}/\lambda_{l}<\infty , \end{array}$$

which proves $c=c^{\prime }$. Meanwhile,

(A12) $$\begin{array}{c} <f\left(x\right),g\left(x\right){>}_{H_{P}}=\sum_{l}f_{l}g_{l}/\lambda_{l},\;\left\{f_{l}\right\},\left\{g_{l}\right\}\in l_{1/\lambda }^{2}. \end{array}$$

The inner products remain equal, so it is also clear that $H_{P}$ is isometrically isomorphic to $l_{1/\lambda }^{2}$. To summarize, we can say that $H_{G}$ and $H_{P}$ are isomorphic by establishing a Hilbert space $l_{1/\lambda }^{2}$ to connect them; or it can be said that PCE and GP with the Mercer kernel generate surrogates in the same Hilbert space. □
